# Intrinsic brain abnormalities in female major depressive disorder patients with childhood trauma: A resting-state functional magnetic resonance imaging study

**DOI:** 10.3389/fnins.2022.930997

**Published:** 2022-08-09

**Authors:** Juran Chen, Qianyi Luo, Yuhong Li, Zhiyao Wu, Xinyi Lin, Jiazheng Yao, Huiwen Yu, Huiqin Nie, Yingying Du, Hongjun Peng, Huawang Wu

**Affiliations:** ^1^Department of Clinical Psychology, The Affiliated Brain Hospital of Guangzhou Medical University, Guangzhou, China; ^2^Department of Radiology, The Affiliated Brain Hospital of Guangzhou Medical University, Guangzhou, China

**Keywords:** childhood trauma, amplitude of low-frequency fluctuation, functional connectivity, middle frontal gyrus, postcentral gyrus, putamen

## Abstract

**Objective:**

Childhood trauma is a strong predictor of major depressive disorder (MDD). Women are more likely to develop MDD than men. However, the neural basis of female MDD patients with childhood trauma remains unclear. We aimed to identify the specific brain regions that are associated with female MDD patients with childhood trauma.

**Methods:**

We recruited 16 female MDD patients with childhood trauma, 16 female MDD patients without childhood trauma, and 20 age- and education level-matched healthy controls. All participants underwent resting-state functional magnetic resonance imaging (MRI). Regional brain activity was evaluated as the amplitude of low-frequency fluctuation (ALFF). Furthermore, functional connectivity (FC) analyses were performed on areas with altered ALFF to explore alterations in FC patterns.

**Results:**

There was increased ALFF in the left middle frontal gyrus (MFG) and the right postcentral gyrus (PoCG) in MDD with childhood trauma compared with MDD without childhood trauma. The areas with significant ALFF discrepancies were selected as seeds for the FC analyses. There was increased FC between the left MFG and the bilateral putamen gyrus. Moreover, ALFF values were correlated with childhood trauma severity.

**Conclusion:**

Our findings revealed abnormal intrinsic brain activity and FC patterns in female MDD patients with childhood trauma, which provides new possibilities for exploring the pathophysiology of this disorder in women.

## Introduction

Major depressive disorder (MDD) is a serious mental disorder that affects mood, interest, and cognitive function (Otte et al., 2016). It has enduring impacts throughout life (Kessler et al., 2007) and heavy economic and social burdens (Murray et al., 2012; Ferrari et al., 2013). Women are approximately twice as likely to experience MDD as men (Seedat et al., 2009). Thus, being a woman is a risk factor for developing MDD (Otte et al., 2016). Researchers have speculated that the sex differences in MDD development might relate to variations in susceptibility (both physical and psychological) as well as environmental factors that work at both the micro and macro levels (Kuehner, 2017). However, the neural mechanisms underlying female MDD patients remain unclear.

Childhood trauma is a common psychological stressor and includes experiences of abuse and neglect (Bernstein et al., 2003). Multiple studies have reported that childhood trauma can predict psychiatric disorders such as bipolar disorder, anxiety, substance use disorder, post-traumatic stress disorder, and MDD (Baldwin et al., 2019; Hailes et al., 2019; McKay et al., 2021). There are sex differences in childhood trauma. Compared with men, the impact of childhood trauma is even more profound in women. Women also have more complex patterns of childhood trauma (Haahr-Pedersen et al., 2020), and the female sex also plays a synergistic role with childhood trauma in certain mental disorders (e.g., anxiety and depressive episodes) (Whitaker et al., 2021). Previous studies of childhood trauma have focused on neuroendocrinology (Silva et al., 2021; Tan et al., 2021), neuroinflammation (Andersen, 2022), and neuroimaging (Tozzi et al., 2020; Ma et al., 2021) to analyze the intrinsic biological mechanisms of MDD. However, trauma-related brain dysfunction is not fully understood. In particular, brain neuroimaging studies of women with childhood trauma experience remain severely lacking. It is, therefore, important to investigate the pathophysiology and etiology of MDD in women who have experienced childhood trauma.

A growing body of evidence indicates that the amplitude of the low-frequency fluctuation (ALFF) method can be used to capture local brain activity and identify various physiological conditions in the brain (Yang et al., 2007; Yan et al., 2009). Previous studies have detected ALFF alterations in MDD with childhood trauma, including in the left insula, right dorsal anterior cingulate cortex, bilateral amygdala, and left orbital/cerebellum (Du et al., 2016; Wu et al., 2020). Moreover, functional connectivity (FC) methods have been developed to measure both the temporal correlations (Du et al., 2016; Wu et al., 2020) and the coordination of brain activity (Biswal et al., 1995; Noble et al., 2019) among multiple brain regions. Yu et al. (2019) reported that childhood trauma across different dimensions of symptoms is associated with abnormal network architecture in patients with MDD. A combination of ALFF and FC has been recommended to investigate abnormal intrinsic brain function in patients with MDD (Hu et al., 2019; Ebneabbasi et al., 2021; Yan et al., 2022). However, ALFF and FC alterations in female MDD patients with childhood trauma have not yet been investigated. To address this gap, we used ALFF and FC methods to explore brain function and FC patterns in female MDD patients with childhood trauma. The aim of this study was to provide new insights into the underlying neurobiological mechanisms of the disease.

## Materials and methods

### Participants

Individual mentalization in early adulthood is not yet fully matured, and individuals in early adulthood are more vulnerable to childhood traumatic experiences (Sonu et al., 2019; Hamlat et al., 2021). Interestingly, the scholar found that early adulthood is the peak period of MDD onset (Kessler et al., 2007). However, mental illness in early adulthood did not raise major attention and age has not been well controlled in previous studies. Thus, we only included female participants in early adulthood (18–35) in this research.

A total of 52 early adulthood women were recruited. The diagnosis of MDD was made by professional psychiatrists referring to the Diagnostic and Statistical Manual of Mental Disorders–Fourth Edition (DSM-V) criteria. The 17-item Hamilton Depression Scale (HAMD) (Zimmerman et al., 2013) was used to measure depression severity. The Childhood Trauma Questionnaire (CTQ) was employed to evaluate the negative impact of childhood trauma. The CTQ can be divided into 5 subscales, including emotional abuse (EA), emotional neglect (EN), sexual abuse (SA), physical abuse (PA), and physical neglect (PN). The cutoff points for the CTQ subscale are as follows: (i) EA ≥ 13, (ii) EN ≥ 15, (iii) SA ≥ 8, (iv) PA ≥ 10, and (v) PN ≥ 10 (Xie et al., 2018; Georgieva et al., 2021). Childhood trauma history was considered to exist in participants scoring over the subscale threshold (moderate–severe). The HAMD and CTQ were only used for the assessment of patients with MDD.

According to the above criterion, participants were divided into MDD with the childhood trauma group (*n* = 16), MDD without the childhood trauma group (*n* = 16), and the healthy control group (*n* = 20). Subjects with MDD were recruited from the outpatient clinics of the Affiliated Brain Hospital of Guangzhou Medical University. We recruited healthy participants from the local community with matching age and education levels. In this study, the exclusion criteria are as follows: (i) any other physical and mental illness except for MDD; (ii) history of seizures, head trauma, or unconsciousness; (iii) received electroconvulsive therapy within the past 6 months, recently taken contraceptives, and taken psychiatric drugs before; (iv) substance dependence; (v) pregnant, lactating, or menstruating women; and (vi) any contraindications to magnetic resonance imaging (MRI). All participants were fully informed and written informed consent was obtained before enrollment. This study was approved by the Ethics Committee of the Affiliated Brain Hospital of Guangzhou Medical University.

### Magnetic resonance imaging data acquisition

The MRI data were obtained on a 3.0T MRI system (Philips, Best, The Netherlands) in the Affiliated Brain Hospital of Guangzhou Medical University. Tampons were used to reduce noise, while foam pads were used to restrain head movement. During the scan, subjects were asked to remain still and close their eyes, but not fall asleep and think. The parameters of the echo plane imaging (EPI) sequence were as follows: repetition time (TR) = 2,000 ms, echo time (ET) = 30 ms, flip angle = 90°, field of view (FOV) = 220 × 220 mm^2^, slices = 33, thickness = 4 mm, inter-slice gap = 0.6 mm, and matrix = 64 × 64. Meanwhile, the parameters of the T1-weighted sagittal images were as follows: TR = 8.2 ms, ET = 3.7 ms, flip angle = 7°, thickness = 1 mm, and matrix = 256 × 256. To strictly control the effect of head movement, we excluded one subject whose head translation was greater than 1.5 mm.

### Magnetic resonance imaging data preprocessing

The fMRI data were conducted by a Data Processing Assistant for Resting-State fMRI Advanced Edition V4.5 (DPARSFA)^1^ (Yan et al., 2016). In addition to the first 10 volumes being removed, all the images were corrected for temporal differences and head motion. We excluded the participants whose image translation movement was more than 1.5 mm or rotational movement was more than 1.5°. The T1-weighted image was co-registered with the average functional image after motion correction. Then, the images were normalized to the Montreal Neurological Institute template and resampled to a spatial resolution of 3 × 3 × 3 mm^3^. Subsequently, the functional images were smoothed with a Gaussian kernel (full-width half-maximum = 4 mm). In addition, in order to remove the effects of nuisance covariates, we regressed headmotor parameters, white matter signals, and CSF signals. Finally, the time series for each voxel was subjected to linear trend reduction and temporal filtering (0.01–0.08 Hz) to reduce low-frequency drift and high-frequency noise (Biswal et al., 1995; Lowe et al., 1998).

### Analysis of amplitude of low-frequency fluctuation

We applied DPASF4.5 to compute ALFF and FC. Briefly, we converted the frequency domain power spectrum of the whole-brain signal with the fast Fourier transform. In addition, based on the power spectrum between 0.01 and 0.08 Hz, we calculated the ALFF. Finally, to minimize variability in general whole-brain ALFF levels between participants, the ALFF value was standardized to the *Z*-value (zALFF).

### Analysis of functional connectivity

Subsequently, a seed-based interregional FC analysis was conducted. Seeds were chosen from brain regions correlated with childhood trauma in between-group ALFF discrepancy. FC analysis was calculated after a time series of the seed area average was extracted. The seed area and the rest of the brain were then correlated voxel-by-voxel. Finally, in order to enhance the normality of the correlation coefficient, we performed a Fisher’s r to z transformation.

### Statistical analysis

Statistical analyses were calculated using Statistical Package for the Social Sciences, version 19.0 (SPSS, Inc., Chicago, United States). Group differences in demographic and clinical data were assessed by one-way analysis of covariance (ANCOVA) or two sample *t*-tests. In this study, significant differences were defined as *p* < 0.05.

To identify the significance of the brain district that had altered ALFF and FC values, a voxel-based ANCOVA was conducted using education and age as covariates. The significance was set with a cluster-level corrected threshold of *p* < 0.05 (cluster-forming threshold at voxel level *p* < 0.001 using the AlphaSim method) (Forman et al., 1995; Poline et al., 1997). Then, to examine group differences in mean ALFF and FC values, two sample *t*-tests were conducted on three groups identified after ANCOVA. Multiple comparison correction was employed with the Bonferroni method, and the significance level was determined at *p* < 0.05/2 = 0.025 (voxel-wise concordance analysis resulting in two significant clusters). In addition, partial correlation analyses were performed to discover the contact of voxel-wise concordance with CTO score in all subjects with MDD [*p* < 0.05/12 = 0.0041 with Bonferroni correction of 12 being due to two clusters and 6 scales (i.e., CTO scale and its 5 subscales)]. Finally, we further explored the association of brain dysfunction with childhood trauma in female patients with MDD by multiple linear regression with CTQ total scores and the characteristic values of brain region (ALFF and FC). Age and education were considered control variables in partial correlation and multiple linear regression.

## Results

### Demographic and clinical measures

As shown in Table 1, no significant difference was found in age and education level among the three groups (all *p* < 0.05). Significant differences were detected in CTQ total score and its subscale scores (e.g., EA, EN, SA, and PN) between MDD with the childhood trauma group and MDD without the childhood trauma group.

**TABLE 1:** Characteristics of MDD with childhood trauma, MDD without childhood trauma, and HC groups.

Characteristics	MDD with childhood trauma (*n* = 16)	MDD without childhood trauma (*n* = 15)	HC (*n* = 20)	F/T	*P*-value
Age, years	24.93 ± 3.73	24.26 ± 3.17	23.40 ± 3.16	0.94	0.39^a^
Education, years	13.93 ± 3.76	14.66 ± 2.76	14.35 ± 1.81	0.26	0.77^a^
HAMD	30.00 ± 6.39	30.20 ± 4.97	−	0.64	0.92^b^
**CTQ score**					
Emotional abuse	11.37 ± 4.67	6.73 ± 1.79	−	3.60	<0.01^b^
Physical abuse	7.00 ± 2.87	6.13 ± 1.55	−	1.03	0.31^b^
Sexual abuse	7.56 ± 4.17	5.26 ± 0.59	−	2.10	0.04^b^
Emotional neglect	18.37 ± 3.11	8.00 ± 2.10	−	10.78	<0.01^b^
Physical neglect	11.31 ± 3.07	5.86 ± 0.91	−	6.59	<0.01^b^
Total	55.62 ± 11.15	32.00 ± 4.32	−	7.67	<0.01^b^

*CTQ, Childhood Trauma Questionnaire; HAMD, Hamilton Depression Rating Scale; plus-minus values are means ± S.D.*

^a^ *The P-values were obtained by one-way analysis of variance test.*

^b^ *The P-values were obtained by two sample-test.*

### Assessed by one-way analysis of covariance plus *post hoc* comparisons of amplitude of low-frequency fluctuation

**FIGURE 1:**
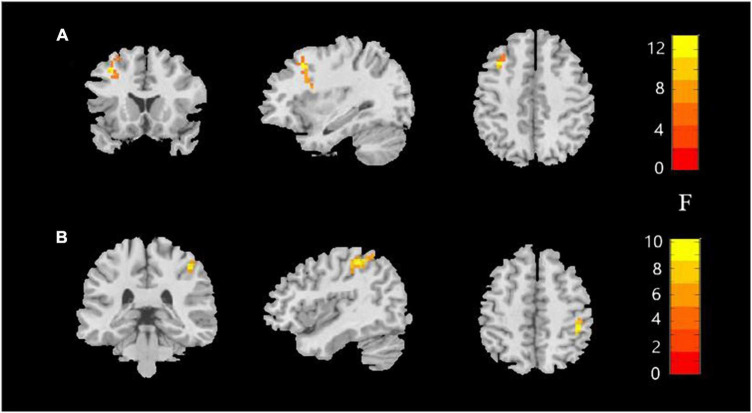
Amplitude of low-frequency fluctuations (ALFF) value among the MDD with childhood trauma, MDD without childhood trauma, and HC groups. One-way ANCOVA with age and education as covariates was performed to compare ALFF maps in the experimental groups. Left middle frontal gyrus **(A)** and right postcentral gyrus **(B)** showed the most significant differences according to ALFF analysis (AlphaSim-corrected *p* < 0.05).

**FIGURE 2:**
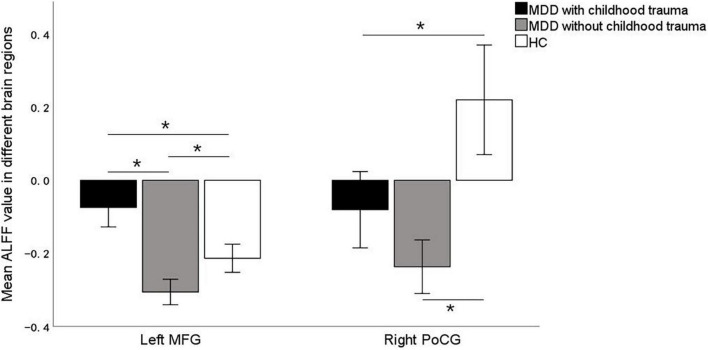
*Post hoc* two samplet—tests were used to determine the between groups differences in ALFF value in left middle frontal gyrus and right postcentral gyrus showing significant differences in ALFF maps in the previous ANOVA. *Bonferroni correction with *P* < 0.025 was set for significance. MFG, middle frontal gyrus; PoCG, postcentral gyrus.

Significant ALFF alterations were found in the left middle frontal gyrus (MFG) (57 voxels) and the right postcentral gyrus (PoCG) (63 voxels) between the three groups (Figure 1 and Table 2). MDD with the childhood trauma group revealed increased ALFF values in the left MFG compared to MDD without the childhood trauma group and the HC group (Figure 2 and Table 3). Moreover, decreased ALFF was detected in the right PoCG in MMD patient groups relative to the HC group. Nonetheless, no significant ALFF difference was discovered among MDD with and without childhood trauma groups (Figure 2 and Table 3).

**TABLE 2:** Group differences in amplitude of low-frequency fluctuations in MDD with childhood trauma, MDD without childhood trauma and HC.

Brain regions	Hemisphere	Peak MNI			Cluster size	F
		X	Y	Z		
Middle frontal gyrus	Left	−33	18	45	57	30.40
Postcentral gyrus	Right	42	−33	51	63	16.35

*MNI, Montreal Neurological Institute; x, y, z, coordinates of primary peak locations in the MNI space.*

**TABLE 3:** Multiple comparisons of ALFF in left middle frontal gyrus and right postcentral gyrus.

Brain regions	Pair group (I VS. J)	Mean difference (I–J)	*P*	95%CI	
Left middle frontal gyrus	G1 VS. G2	0.2311	<0.001	0.1567	0.3054
	G1 VS. G3	0.1389	<0.001	0.0695	0.2082
	G2 VS. G3	−0.092	<0.001	−0.1628	−0.0215
Right postcentral gyrus	G1 VS. G2	0.1544	0.242	−0.0603	0.3692
	G1 VS. G3	−0.3008	0.002	−0.5013	−0.1003
	G2 VS. G3	−0.4552	<0.001	−0.6594	−0.2511

G1, MDD with childhood trauma; G2, MDD without childhood trauma; G3, HC.

### Functional connectivity analyses

**FIGURE 3:**
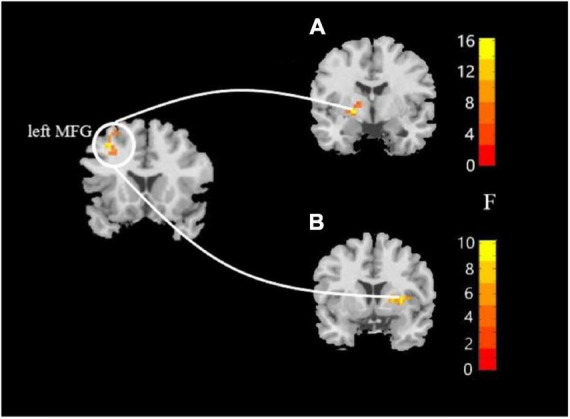
Resting-state functional connectivity analyses among MDD with childhood trauma, MDD without childhood trauma, and HC groups. One-way ANCOVA with age and as covariates was performed to compare functional connectivity maps in all the three groups and identified significant differences between left middle frontal gyrus and bilateral putamen (**A**, left putamen gyrus; **B**, right putamen gyrus) (AlphaSim-corrected *p* < 0.05).

**FIGURE 4:**
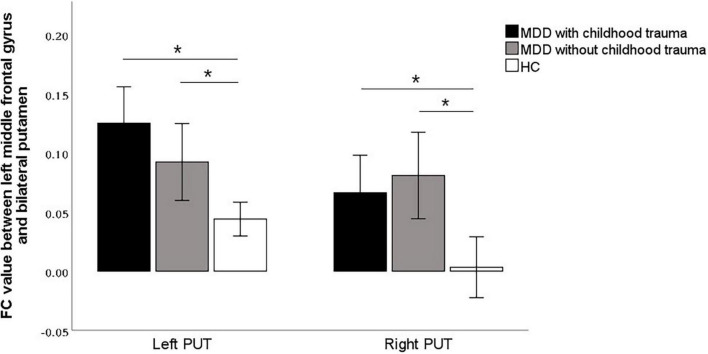
*Post hoc* analyses revealed significant increased functional connectivity of left PUT to left middle frontal gyrus in MDD with childhood trauma compared to both MDD without childhood trauma and HC group. The functional connectivity of right PUT to left middle frontal gyrus in the MMD patient groups were significantly higher than that in the HC group, but there was no significant difference between those with and without childhood trauma. *Bonferroni correction with *P* < 0.025 was set for significance.

In this study, the brain areas that showed group differences in the ALFF analysis were selected as seed (i.e., left MFG) in the FC analysis. Significant FC difference in the left MFG–bilateral putamen was observed between groups (Figure 3 and Table 4). Compared to the HC group, MDD groups showed increased FC between left MFG and bilateral putamen (Figure 4 and Table 5). However, no observable discrepancy was found between MDD with and without childhood trauma (Figure 4 and Table 5).

**TABLE 4:** FC differences between left middle frontal gyrus seed and left putamen gyrus and right putamen gyrus in MDD with childhood trauma, MDD without childhood trauma, and HC.

Seed	Brain regions	Peak MNI			Cluster size	*F*
		X	Y	Z		
Left middle frontal gyrus	Left putamen gyrus	−21	−3	−3	74	12.41
	Right putamen gyrus	30	6	3	81	8.57

MNI, Montreal Neurological Institute; x, y, z, coordinates of primary peak locations in the MNI space.

**TABLE 5:** Multiple comparisons of functional connectivity between left middle frontal gyrus seed and left putamen gyrus and right putamen gyrus.

Seed	Brain regions	Pair group (I VS. J)	Mean difference (I–J)	*P*	95%CI	
Left middle frontal gyrus	Left putamen gyrus	G1 VS. G2	0.0328	0.207	−0.0109	0.0766
		G1 VS. G3	0.0810	<0.001	0.0401	0.1219
		G2 VS. G3	0.0482	0.018	0.0065	0.0898
	Right putamen gyrus	G1 VS. G2	−0.0145	1.000	−0.0677	0.0387
		G1 VS. G3	0.0629	0.009	0.0132	0.1126
		G2 VS. G3	0.0774	0.001	0.0268	0.1281

G1, MDD with childhood trauma; G2, MDD without childhood trauma; G3, HC.

### Partial correlation analyses and multiple linear regressions analyses

**FIGURE 5:**
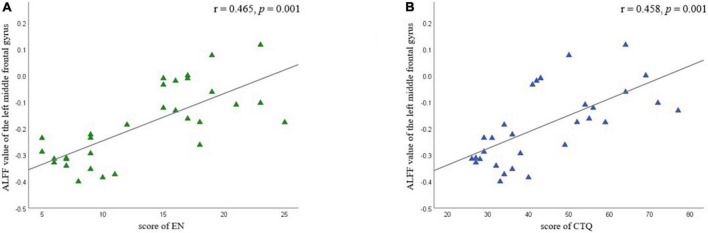
Partial correlation analyses between amplitude of low-frequency fluctuation value (ALFF) in left middle frontal gyrus and childhood trauma scores in different subscale were performed by combining all of MDD participants as a whole. Age and education were considered as control variables. **(A)** Shows the correlation between EN scores and ALFF value of the left middle frontal gyrus; **(B)** shows the correlation between CTQ total scores and ALFF value of the left middle frontal gyrus. EA, emotional neglect.

Partial correlation analysis confirmed the positive correlation between ALFF in the left MFG and EN scores (*r* = 0.465 *p* = 0.001) and CTQ total score (*r* = 0.458, *p* = 0.001), respectively (Figure 5 and Table 6). We did not find a significant correlation between FC and CTQ total scores or its subscale scores. Regression analyses further showed the correlation of ALFF value in the left MFG on childhood trauma (*F* = 2.476, *p* < 0.05) (Table 7).

**TABLE 6:** Partial correlation between CTQ scores and ALFF.

Brain regions	Emotional abuse		Physical abuse		Sexual abuse		Emotional neglect		Physical neglect		Total score of CTQ	
	*r*	*P*	*r*	*P*	*r*	*P*	*r*	*P*	*r*	*P*	*r*	*P*
Left middle frontal gyrus	0.262	0.068	0.091	0.533	0.352	0.013	0.465*	0.001	0.331	0.020	0.458*	0.001
Right postcentral gyrus	−0.009	0.949	−0.018	0.903	0.025	0.865	0.142	0.329	0.236	0.102	0.116	0.426

ALFF, amplitude of low-frequency fluctuations; CTQ, Childhood Trauma Questionnaire.

Age and education were considered as control variables.

*p_adj_ < 0.004, corrected for multiple comparisons.

**TABLE 7:** Multiple linear regressions analyses between childhood trauma and brain dysfunction.

	*R* ^2^	*F*	*B*	*T*
**Step1**				
Age	0.048	1.22	0.136	0.885
Education			−0.236	−1.534
**Step2**				
Age	0.204*	2.476*	−0.009	−0.055
Education			−0.142	−0.98
ALFF of left MFG			0.452	3.115*
ALFF of right PoCG			0.026	0.176
FC of right PUT			−0.084	−0.433
FC of left PUT			0.049	0.253

*p < 0.05.

## Discussion

In this study, we focused on ALFF and FC alterations in female MDD patients with childhood trauma. There was increased ALFF in the left MFG and right PoCG in MDD with childhood trauma compared with MDD without childhood trauma. The brain regions with significant ALFF discrepancies were selected as seeds for the FC analyses. There was increased FC in the left MFG and bilateral putamen gyrus. Moreover, we confirmed an association between altered ALFF and childhood trauma history. Together, our findings indicate the presence of abnormal intrinsic brain activity and FC patterns in female MDD patients with childhood trauma. The results of our research also offer important insights into the neurobiological mechanisms of MDD and childhood trauma.

An interesting finding in this study was that, in the left MFG, ALFF was higher in the MDD group with childhood trauma than in the group without childhood trauma. In addition, ALFF was positively correlated with CTQ scores and EN. Previous studies have reported that left MFG activation is associated with working memory (Zhang et al., 2003), the processing of social information and social perception (Vollm et al., 2006), memory retrieval (Tulving et al., 1994), and emotion regulation (Ochsner and Gross, 2005; Bermpohl et al., 2006). This area is also associated with rumination (Wang et al., 2018), which in turn increases an individual’s risk of MDD (Abela and Hankin, 2011). For example, Shors et al. (2017) reported that interventions targeting rumination generally reduce MDD incidence in women. Furthermore, O’Mahen et al. (2015) revealed that EN and abuse in childhood are associated with depression, with rumination partially mediating this effect. To some extent, our findings support this conjecture. Local brain activity may be affected by previous childhood trauma, especially EN, which in turn affects cognitive processing patterns such as rumination. Notably, Tulving et al. (1994) reported that the left MFG is related to memory retrieval. Thus, abnormal left MFG activation in individuals with childhood trauma may repeatedly trigger traumatic memories and exacerbate rumination. This may be the underlying cause of heightened depressive symptoms in MDD patients with childhood trauma. Overall, our results suggest that abnormal left MFG function might indicate the impact of childhood trauma in young adult women with MDD.

We also revealed that patients with MDD were at a higher risk for dysfunction in the right PoCG; however, there were no significant differences between patients with and without childhood trauma. Numerous studies have noted that the PoCG is mainly involved in the processing of some sensory information (Phillips et al., 2003), cognitive activities (Wager and Smith, 2003), and emotional processing (Luo et al., 2022). Tadayonnejad et al. (2015) reported that regional properties of neural activity in the PoCG are associated with depression severity. Moreover, neuroimaging studies have demonstrated structural and functional changes in the precentral and postcentral gyri of patients with MDD (Guo et al., 2011; Wang et al., 2012). Abnormal brain function in the PoCG may thus be a unique neurobiological feature of MDD; our results support this idea. Together, these findings provide theoretical support for further research into the relationship between the PoCG and MDD.

In this study, we investigated FC patterns in female MDD patients with childhood trauma. We measured the FC of each cluster vs. the rest of the brain using altered ALFF clusters with clinical correlations as the ROIs. The FC between the left MFG and bilateral putamen was observably increased in the MDD group compared with the HC group. The putamen is associated with motor control and learning (Luo et al., 2020), is one of the core regions for emotion production and processing (Wager et al., 2003), and plays an important role in cognitive and executive functions (Peters et al., 2016). Su et al. (2018) reported that a decrease in glucose metabolism in the putamen of patients with MDD impaired FC to key centers, such as the inferior and middle frontal gyri. Although the results of our study differed from those of predecessors, the discrepancies may be caused by differences in sample size, research subjects, or other reasons. Abnormal connectivity between the MFG and the putamen appears to be an important characteristic of MDD. However, our study revealed that the FC between the left MFG and bilateral putamen had no observable discrepancy in the MDD with the childhood trauma group compared with the MDD without the childhood trauma group. Jeong et al. (2021) found that trauma exposure may be related to structural alterations in the MFG and putamen. Thus, trauma exposure may also be an important factor underlying structural abnormalities of the MFG and putamen, but we need further research to find out if there are also functional abnormalities in this brain region. Furthermore, both trauma exposure and MDD appear to be associated with these two cognitively related regions. Thus, perhaps the main crux of depression with childhood trauma is changing in cognition; this may have a certain guiding significance for the clinical treatment of MDD and will be a major direction of our future research. Collectively, our findings provide an important base for investigating the neuropathological mechanisms of MDD as well as those of childhood trauma.

Our study has certain limitations. First, it remains unclear whether self-reported trauma history reflects authentic experiences during childhood and early adolescence. To minimize information bias, we, therefore, conducted in-depth interviews to confirm adverse childhood experiences. In addition, the study age was set at early adulthood (18–35 years of age) to minimize any differences in the duration of childhood trauma. However, this study did not further subdivide the types of childhood trauma. This is also the direction of future research to further analyze the effects of childhood trauma on the brain in terms of different dimensions, intensities, and durations, for example. Second, this study used a cross-sectional approach with small sample size and lacked any comparisons with the male population. The current results should, therefore, be interpreted with caution. Future studies will expand the sample size to further validate the results and add a male control group to investigate whether the identified brain regions are unique to women.

## Conclusion

Overall, after controlling age-related confounding factors as much as possible, our study found that left MFG abnormality and left MFG–putamen dysfunction may be unique neural mechanisms in female MDD patients with childhood trauma. Our findings provide a basis for future research into the relationship between childhood trauma and MDD.

## Data availability statement

The raw data supporting the conclusions of this article will be made available by the authors, without undue reservation.

## Ethics statement

The studies involving human participants were reviewed and approved by The Affiliated Brain Hospital of Guangzhou Medical University. The patients/participants provided their written informed consent to participate in this study.

## Author contributions

HP, HW, and JC designed the experiments. YL, ZW, XL, JY, HY, HN, and YD performed the clinical data collection and assessment. JC and QL performed the neuroimaging data analysis and wrote the draft. All authors discussed the results and reviewed the manuscript.

## Footnotes

1
www.restfmri.net/forum/DPARSF
